# Supplementary material for: Synergistic Effect of Propolis and Antibiotics on Uropathogenic Escherichia coli
Source: Antibiotics (Basel). 2020 Oct 27;9(11):739. doi: 10.3390/antibiotics9110739 (PMC7692270; doi:10.3390/antibiotics9110739)
Supplement: Supplementary file 1 [file antibiotics-09-00739-s001.pdf]

Supplementary data

Table S1. MICs of antibiotics, propolis (0.5x MIC) and both for a panel of UPEC. All the data are determined in mg/L. The different thresholds were: ofloxacin (OFX), R>0.5 mg/L; ceftriaxone (CRO), R>2 mg/L; ertapenem (ETP), R > 1 mg/L; fosfomycin (FOS), R>32 mg/L) [24].

Strains	Resistance	Ofloxacin	Ofloxacin + Propolis	Ceftriaxone	Ceftriaxone + Propolis	Ertapenem	Ertapenem + Propolis	Fosfomycin	Fosfomycin + Propolis	Propolis
CFT073	Sensitive	0.25	0.06	0.5	0.125	0.06	<0.03	8	4	256
NECS892841	Sensitive	0.5	0.5	1	0.125	0.06	0.03	512	256	256
NECS30990	Sensitive	0.25	0.125	0.5	0.06	0.03	<0.03	8	8	128
NECS858785	OFX R	2	01	0.25	<0.06	0.06	<0.03	128	128	256
NECS864598	OFX R	32	8	0.5	0.06	0.125	0.06	512	256	256
NECS892420	BLSE	>32	16	>32	2	0.25	0.06	8	8	256
NECS118564	BLSE	>32	32	>32	2	0.5	0.125	8	8	256
